# Sticking for a Cause: The Falciparum Malaria Parasites Cytoadherence Paradigm

**DOI:** 10.3389/fimmu.2019.01444

**Published:** 2019-06-27

**Authors:** Wenn-Chyau Lee, Bruce Russell, Laurent Rénia

**Affiliations:** ^1^Singapore Immunology Network (SIgN), Agency for Science, Technology and Research (A^*^STAR), Singapore, Singapore; ^2^Department of Microbiology and Immunology, University of Otago, Dunedin, New Zealand

**Keywords:** malaria, *Plasmodium*, cytoadherence, pathogenesis, host immune responses

## Abstract

After a successful invasion, malaria parasite *Plasmodium falciparum* extensively remodels the infected erythrocyte cellular architecture, conferring cytoadhesive properties to the infected erythrocytes. Cytoadherence plays a central role in the parasite's immune-escape mechanism, at the same time contributing to the pathogenesis of severe falciparum malaria. In this review, we discuss the cytoadhesive interactions between *P. falciparum* infected erythrocytes and various host cell types, and how these events are linked to malaria pathogenesis. We also highlight the limitations faced by studies attempting to correlate diversity in parasite ligands and host receptors with the development of severe malaria.

## Introduction

Malaria continues to be a significant healthcare problem to many human populations, despite efforts to eliminate this debilitating and potentially fatal tropical disease. While the malaria mortality did not significantly change between 2015 and 2016, the number of malaria cases increased by five millions within the same period (1). Among the medically important malaria parasites (2, 3), *Plasmodium falciparum* is the primary cause of severe disease and death (4, 5).

**Figure 1:**
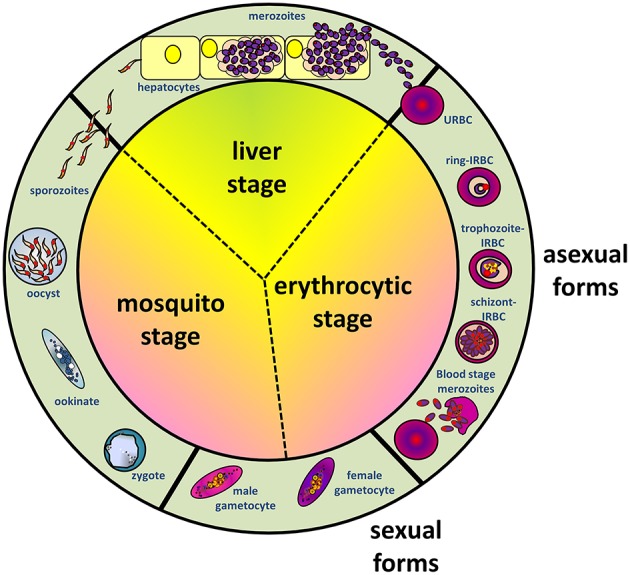
Schematic diagram depicting life cycles of *Plasmodium falciparum*, involving *Anopheles* mosquito and human hosts, where the stages in humans can be furthered divided into liver (exoerythrocytic) and erythrocytic stages.

As with other malaria parasites, *P. falciparum* has a complex life cycle involving humans as the intermediate host and *Anopheles* mosquitoes as the definitive host (where sexual reproductive forms of the parasites establish) (Figure 1). During its blood meal, the infected female *Anopheles* mosquito releases *Plasmodium* sporozoites from its salivary glands into the dermis of human host. A proportion of sporozoites migrate rapidly to the blood capillaries, then to the liver and invade the parenchymal hepatocytes after traversing the Kupffer cells (6). Inside the invaded parenchymal cells, parasites asexually multiply, producing numerous (~20,000–40,000) liver merozoites. Subsequently, these merozoites are released into the blood circulation, where they target and invade the erythrocytes (RBCs). It is the erythrocytic life cycle that is responsible for the manifestation of signs and symptoms in malaria. Within the infected erythrocytes (IRBCs), the blood stage-parasites develop from the early ring forms into trophozoites, subsequently form schizonts, which upon maturation will rupture and release blood stage merozoites to invade other uninfected erythrocytes (URBCs). Meanwhile, a fraction of the parasites are driven into the formation of sexual forms (gametocytes), which will be taken up by mosquitoes during feeding. Inside the mosquito, fertilization of male and female gametocytes leads to zygote formation. Subsequent developments lead to formation of salivary gland sporozoites, which are infective to the human host.

One fascinating aspect of *P. falciparum* infection is the cytoadherence phenomenon associated with the late stage-IRBC (7), which is considered to be a major contributor to the pathogenesis of falciparum malaria (8). As the parasite develops and matures within the host RBC, it causes substantial alteration to the IRBC membrane architecture, which changes various rheological properties of the IRBC, including its cytoadhesive characteristics (9–11). Here, we review the different types of cytoadhesive interactions of the IRBCs, how they are linked to each other, the molecular and cellular mechanisms behind these phenomena and their proposed involvement in malaria pathology. We also discuss knowledge gap, controversies and diverging views on the role of cytoadherence in *P. falciparum* immunopathogenesis.

## Complex Profile of *P. falciparum*-IRBC Cytoadherence

The cytoadherence of IRBCs to host cells in falciparum malaria is highly complex, involving at least three distinct groups of parasite-derived variant surface antigens (VSAs) encoded by multigene families, namely; *P. falciparum* Erythrocyte Membrane Protein 1 (PfEMP1) (12, 13), Subtelomeric Variable Open Reading frame (STEVOR) proteins (14), and repetitive interspersed family (RIFIN) proteins (15). The temporal expression of these ligands also differs, with PfEMP1 being expressed the earliest (transcription starts at ring forms and protein surface expression happens when parasites mature into trophozoites) (16, 17), followed by RIFIN (17, 18), then STEVOR (17, 19–21). In addition, only few members of VSA are expressed by a single IRBC. Members of these VSA families bind to a wide range of different host-derived proteins, proteoglycans, and glycosaminoglycans (as summarized in Table 1). The role of RIFINs and STEVORs in the cytoadhesion of *P. falciparum* IRBCs is undoubtedly of significance. Apart from forming rosettes (a cytoadherence phenomenon by the late stage-IRBCs, which is elaborated in latter section) via interactions with the A antigens on the URBCs (15), some RIFINs can interact with leukocyte immunoglobulin-like receptor B1 (LILRB1), which inhibits the activation of B cells and natural killer (NK) cells expressing LILRB1 (51). This discovery suggests the involvement of RIFIN in the parasite's immune-evasion mechanisms. STEVOR proteins interact with the RBCs, and current evidence suggests their involvement in immune evasion, rosette formation and merozoite invasion (14, 21). By comparison, PfEMP1 binds to diverse array of host receptors on different host cells, leading to suggestions of its involvement in immune evasion (52) and immune modulation (53). Hence, it is generally accepted that the PfEMP1 is the most important of the VSAs. A detailed description of PfEMP1 variant domains and their binding targets, as well as the switching of expressions, have been elegantly reviewed elsewhere (54–56). In general, the extracellular domain of PfEMP1 can be classified into four major regions, which are the N-terminal segment (NTS), the C2 region, the Cysteine-rich inter-domain region (CIDR), and the Duffy binding-like region (DBL). These regions are responsible for the diverse cytoadherence phenomena attributed to PfEMP1, and difference in these regions gives rise to different cytoadherence properties, hence different tissue tropism for different strains of parasites (57, 58). Importantly, the various parasite-derived antigens that are expressed on IRBC surface make IRBCs an obvious target for host's immune system (59).

**Table 1:** Host-derived receptors for *P. falciparum* cytoadherence ligands.

**Ligands**	**Receptors**	**Expression sites**	**References**
PfEMP1	Complement receptor 1 (CR1/CD35)	RBCs, leukocytes, splenic follicular dendritic cells	(22)
	Chondroitin sulfate A (CSA)	Endothelial cells, placental syncytiotrophoblasts	(23, 24)
	Hyaluronic acid (HA)	Placenta, and other connective, epithelial and neural tissues	(25)
	Heparan sulfate (HS)	All tissues	(26, 27)
	Platelet glycoprotein 4 (CD36)	Platelets, RBCs, monocytes, differentiated adipocytes, microdermal endothelial cells, skeletal muscles	(28–33)
	Intercellular adhesion molecule 1 (ICAM1/CD54)	Endothelial cells, leukocytes	(29, 31, 33–36)
	Vascular cell adhesion protein molecule 1 (VCAM1/CD106)	Endothelial cells	(31, 33, 35)
	RBC group A/B antigens	RBCs	(37, 38)
	Platelet endothelial cell adhesion molecule 1 (PECAM-1/CD31)	Platelets, monocytes, neutrophils, T-cells, endothelial cell (intercellular junctions)	(39, 40)
	Ig M	Circulation	(41–43)
	P-selectin (CD62P)	Activated platelets, activated endothelial cells	(31, 33, 44)
	E-selectin (CD62E)	Activated endothelial cells	(29, 35)
	Endothelial protein C receptor (EPCR/CD201)	Endothelial cells	(45–47)
	Hyaluronan-binding protein 1 (HABP1/gC1qR/P32)	Extracellular matrix, endothelial cells, platelets	(48, 49)
	Neural cell adhesion molecule (NCAM)	Endothelial cells	(50)
STEVOR	Glycophorin C (Gly C)	RBC	(14)
RIFIN	RBC group A antigen	RBC, B cells, NK cells	(15, 51)

The IRBC-cytoadherence events are usually classified based on their binding sites, i.e., endothelial cytoadhesion, cytoadhesion to placental syncytiotrophoblasts, platelets, URBCs (rosetting phenomenon) and leukocytes (monocytes, macrophages, and dendritic cells) (23, 60–62). *P. falciparum* IRBCs can adhere to each other through platelet bridges, forming aggregates of IRBCs, a mechanism defined as autoagglutination (63–65). This phenomenon has been shown to be uncorrelated to rosetting and parasitemia, but significantly associated with severe malaria (63). These different interactions between the parasites and the host described above have been proposed to shape the immunopathobiology of malaria.

## Why do *P. falciparum* IRBC Cytoadhere?

Within hours after the *P. falciparum* merozoite invading the RBCs, the relatively low intra-erythrocytic viscosity of liquid hemoglobin is transformed into viscous gel-like cytoplasm of a developing IRBC (66). Besides, the parasite also remodels the IRBC by building a trafficking network with its parasite-derived proteins and organelles (such as Maurer's cleft) to bring in nutrients essential for its survival (67). The net consequence of these modifications (~10 h post-invasion) is a host cell with a compromised rheological profile (68). Such biomechanical changes render the IRBCs highly susceptible to splenic filtration. Within a spleen, the sinusoids of the red pulps act as a mechanical filter of the circulation. All entities in circulation have to move through the narrow (4 μm at its widest point) inter-endothelial slits (IES) of the red pulps (69). These are the smallest passage space for the blood circulation (69, 70). Healthy erythrocytes with normal morphology and rheology will be able to move through these IES whereas the abnormal cells will be retained and engulfed by the macrophages. As the red pulp of spleen is very effective in destroying rheologically impaired and less deformable erythrocytes, the developing malaria parasite has developed mechanisms that alters the host cell in some ways to escape splenic clearance (71). To this end, *P. falciparum* IRBCs avoid splenic clearance by cytoadhering to the vascular endothelium and sequestering in capillary beds of organs that are less dangerous than the spleen (72).

The central role of the spleen as a selective pressure for the evolution of cytoadhesive IRBCs is supported by the fact that in falciparum malaria patients and *P. falciparum*-infected monkeys whose spleens were removed prior to the infection, late stage-IRBCs that do not sequester are readily detected in peripheral blood (73–75). These circulating late-stage IRBCs have lost the capacity to cytoadhere to endothelial cells (74). These observations form the basis for the development of an anti-sequestration vaccine against *P. falciparum* (76). Theoretically, under spleen-intact conditions, the blockade of late stage-IRBCs to cytoadhere to endothelial cells will render these forms highly susceptible to splenic filtration. Thus, IRBC-cytoadherence plays critical roles in the immune-escape strategies by *P. falciparum*.

Besides endothelial cells, late stage-IRBCs can also adhere to URBC, forming flower-like structures known as “rosettes” (61). To date, *P. falciparum* rosetting has been attributed to three ligands, namely PfEMP1, STEVOR, and RIFIN (11, 13–15). Various host-derived receptors on RBCs have been found to be rosetting receptors (Table 1), the majority of these interact with the variant extracellular domains of PfEMP1. The binding affinity of these variants to the various receptors depends on the sequences coded for these regions, which has been described in detail elsewhere (55, 57, 77). Various roles have been proposed for rosetting; firstly, to facilitate merozoite invasion by bringing URBCs closer to the intracellular parasite. However, this “invasion facilitation” hypothesis for rosettes has been ruled out (78). The second proposed role for rosetting is that URBCs mask parasite-derived antigens (VSAs) expressed on the surface of IRBCs, allowing them to escape immune-recognition by antibodies or phagocytes. Practically, this masking strategy is similar to those applied by other parasites such as the blood flukes *Schistosoma* spp., where the flukes adsorb host-derived antigens (such as the blood surface A, B, H, Lewis b+ antigens) onto its surface (79–81). During the course of malaria infection, phagocytosis of IRBC plays a critical role in the clearance of parasites, especially in the spleen as mentioned earlier (82, 83). Opsonization of IRBCs leads to phagocytosis by the host phagocytes. The opsonization of IRBCs happens via antibody-mediated recognition and complement deposition (84–87). For instance, complement-decorated IRBCs are opsonized through the complement receptor 1 (CR1/CD35) (86, 88). Interestingly, CR1 on URBC is a receptor used by PfEMP1 on the surface of *P. falciparum-*IRBC in rosetting (22). Formation of rosette via CR1 may block this phagocytosis pathway. Meanwhile, phagocytosis can also be mediated in a complement-independent, CD36-dependent manner (89, 90). Likewise, CD36 is also one of the receptors that bind with PfEMP1 for rosette formation (28) (Table 1). Although direct evidence of rosette hampering phagocytosis has yet to be reported, a previous study has demonstrated an inverse relationship between the amount of group A antigens (another rosetting receptor) being expressed on IRBC and its susceptibility to phagocytosis (91), and this may be linked to the better ability of blood group A-IRBCs to form rosettes (92). In addition, the larger size of a rosette relative to individual IRBCs may be more difficult to be engulfed by individual phagocytes as well. Previously, it has been demonstrated that opsonized targets larger than 3 μm and non-opsonized targets larger than 2 μm negatively affect the attachment step of phagocytosis (93). Of note, the thickest point of a RBC is 2–2.5 μm whereas the thinnest point of this cell is ~1 μm. Thus, size wise, a rosette will affect at least this critical step of phagocytosis. Furthermore, adherence of an IRBC to a URBC significantly reduces the deformability of the whole rosetting structure further, as compared to a non-rosetting IRBC harboring parasite of similar stage (68, 94). Such larger, more rigid yet stable structures are likely to be “mechanically” sequestered in microvasculature and may not even be able to reach the spleen.

Apart from endothelial cytoadhesion and rosetting as described above, some strains of *P. falciparum* can sequester within the placental intervillous space of pregnant patients (95), particularly the first-time-pregnant mothers (96). This enables the parasite to escape maternal immune responses (97). Interestingly, parasites that can sequester in placenta usually do not form rosettes well (98). On top of these evasion mechanisms, there are reports showing ability of PfEMP1 and RIFIN to modulate or suppress the host's immune responses as mentioned earlier (51, 53). Thus, it seems that *P. falciparum* uses various cytoadherence phenomena as an immune-escape mechanism.

## Host Immune Responses and Antigenic Variation of the Cytoadherence Ligands

Since the cytoadherence of IRBCs relies on the IRBC-surface expression of parasite-derived cytoadherence ligands, these ligands would be easily recognized and hence destroyed by the host's immune system (59, 99). For instance, antibodies against PfEMP1 have been shown to inhibit rosette formation and induce phagocytosis in experiments using a laboratory-adapted *P. falciparum* strain (100). Antibodies raised against STEVOR expressed by different stages of *P. falciparum* can also inhibit either rosetting or merozoite reinvasion (14). Furthermore, the level of antibodies specific for RIFINs in pediatric malaria patients was reported to be positively correlated with the speed of parasite clearance (101). In fact, antibodies targeting the VSA have been shown to confer protection against malaria (101–106). For an extra level of survival advantage to the parasites, these critical cytoadhesion ligands are VSA coded by multigene families as mentioned earlier (107). During multiplication, VSA expression changes, with a fraction of the progeny expressing a different set of VSAs. Such switching of VSA expression hampers the successful development of an immune response against all IRBCs (108–110). Taking PfEMP1 as an example, DBL and CIDR are the two regions of its extracellular domain responsible for the most of its cytoadherence activities (55, 111). Following the expression switching, the extracellular domains of PfEMP1, hence the binding receptors (targets) are different (112). Nevertheless, many binding receptors targeted by various PfEMP1 extracellular domains are available on endothelial cells. This also partly explains the diverse cytoadhesion receptors for PfEMP1, where the sequestration of IRBCs continues even with altered PfEMP1 variant expression.

## Side Effects of the Parasite Sequestration Escape Strategy

While the evolution of cytoadhesive IRBCs by *P. falciparum* has proven to be a potent immune-evasion strategy, the sequestration of IRBCs has an important side effect, which is the development of severe malaria (113). The manifestation of severe malaria largely depends on the site of sequestration. For instance, cytoadhesion of the IRBCs to syncytiotrophoblasts causes placental malaria, which is characterized by the inflammation of placental tissues, occlusion of nutrient supply to the fetus by the mother, resulting in higher risk of premature delivery, low birth weight of the neonates and subsequent negative impacts on future growth and development (114, 115).

Cytoadhesion of IRBCs to endothelial cells directly activates the endothelial cells, as shown *in vivo* (116) and *in vitro* (117), which in part may lead to endothelial injuries and vascular leakage (118). Various studies have implicated PfEMP1 [particularly its interaction with endothelial protein C receptor (EPCR)] in the pathogenesis of cerebral malaria, one of the most important forms of malaria-induced complications (45, 119–121). Nevertheless, the definitive *in vivo* demonstration of its involvement remains to be performed. There are apparent differences between the *in vitro* and *in vivo* conditions, encompassing content of nutrients, waste products, hormones, cytokines, oxygen level and shear force to name a few, as highlighted elsewhere (122). These differences may become the confounding factors in *in vitro* studies. However, the advancement of technology in the *in vivo* vascular imaging may provide a platform for the relevant *in vivo* works in future (123).

Based on the available information, a simplified sequential development of PfEMP1-mediated cerebral malaria has been suggested (7). The series of parasite-host interactive events start with the IRBCs binding to the endothelial cells via EPCR (119). EPCR plays protective role in maintaining the integrity of circulation through its ability to activate protein C, which is anti-coagulative and anti-inflammatory. The binding of IRBC-PfEMP1 to EPCR may hamper the protein C activation by EPCR, hence reducing the level of activated protein C in the microvasculature affected, which facilitates thrombin formation (112). Such pro-coagulative environment further contributes to compromising microvasculature integrity. Following this, endothelial activation and inflammation may happen (124). The early onset of endothelial inflammation is characterized by the release of Weibel-Palade bodies and subsequent endothelial surface expression of P-selectin and von Willebrand factor (vWF) (113, 125, 126), which in turn mediate leukocyte and platelet rolling on inflamed endothelial cells (127).

Weibel-Palade bodies are the storage granules of endothelial cells (128). This structure contains of a number of components (P-selectin, VWF, angiopoietin 2, IL-8) that have been associated with endothelial injuries and vasculature leakage in malaria pathogenesis (113, 125, 126, 129–134). Other reported components of Weibel-Palade bodies include eotaxin 3, CD63, tissue plasminogen activator (TPA), factor VIII, endothelin 1, osteoprotegerin (OPG), alpha-(1,3)-fucosyltransferase (FUT6), endothelin-converting enzyme, calcitonin gene-related peptide, and insulin-like growth factor-binding protein 7 (IGFBP7). These components are involved in various homeostasis and inflammation related functions encompassing vasculature toning, inflammation and repair, regulating blood coagulation and angiogenesis (135–143). Remarkably, the release of different components within Weibel-Palade bodies is tightly regulated according to the microenvironment of the vasculature (140, 144, 145). This enables the endothelial cells to respond to changes of its microenvironment such as injuries, inflammation or shear stress changes. For instance, the release of VWF from Weibel-Palade bodies by endothelial cells can be triggered by interruption of blood flow (146). In such pro-coagulation environment, platelets can also serve as the bridge between IRBCs and endothelial cells, allowing cytoadhesion to happen even on endothelial cells devoid of principal cytoadhesion receptors (147). Additionally, platelet-mediated autoagglutination of IRBCs may happen in parallel (63), which further disrupts blood flow and activates the endothelial cells (148). Furthermore, angiopoietin-2 released from Weibel-Palade bodies can disrupt the integrity of endothelial junctions, which drives vasculature leakage (149). Following the “first bout” of endothelial inflammation, the expression of EPCR and thrombomodulin by host endothelial cells is downregulated (150), aggravating the pro-coagulation situation. The subsequent release of cytokines triggers expression upregulation of endothelial cell adhesion molecules (CAMs) such as ICAM1, E-selectin, and VCAM (151, 152). ICAM1 is used by other IRBCs to remain sequestering in the microvasculature, possibly with an expression switch of PfEMP1 variants (7). Notably, the disrupted blood flow can cause metabolic acidosis, which further facilitates the acidic pH-dependent binding of IRBCs to receptors like ICAM1 and CD36 (153). The vicious cycle continues, and the integrity of blood brain barrier is altered, leading to hemorrhages and possibly death if left without proper medical intervention.

The hypothesized sequences of pathological events described above remain to be validated fully. Of note, the dual EPCR/ICAM1 binding ability by certain PfEMP1 variants has been demonstrated (154), which may confound the hypothesized sequences of vascular pathogenesis events. Nevertheless, the critical role of EPCR in severe malaria pathogenesis has been highlighted by recent studies. The EPCR-binding *P. falciparum* isolates have been shown to be associated with severe malaria in both adults and children, with different clinical presentations including cerebral malaria, retinopathy and severe malaria-induced anemia (45–47, 155–159). On the other hand, falciparum malaria cases with predominantly CD36-binding parasites have been correlated with uncomplicated clinical presentations (159). Succinctly, the complex IRBC cytoadherence events trigger biological cascade reactions that lead to severe malaria pathologies.

## *P. falciparum* Rosetting and Severe Disease

Rosetting was first reported in the simian malaria parasite *P. fragile*, and subsequently in *P. falciparum* and all other human malaria parasites (61, 160, 161). While it has been suggested that rosetting may aggravate the vasculature occlusion initiated by endothelial-cytoadhered IRBCs (162–164), its importance to pathogenesis of falciparum malaria is still debated. Associations between rosetting rates and malaria severity have been confounded with locality. African cohorts showed positive correlation between rosetting and malaria severity, where association of rosetting rates with parasitemia and different clinical parameters of severe malaria, as well as correlation between malaria severity and impairment of rosette formation due to availability of anti-rosette antibodies in serum and genetic blood disorders with abnormal erythrocytes have been reported (165–169). On the other hand, those conducted in Asia could not find such correlation (170, 171). Although correlation-based findings help to generate hypotheses, it is also important not to overlook the availability of confounding factors in many correlation studies, and the difference between a correlation and a causation.

As mentioned earlier, PfEMP1 is one of the key rosetting ligands for *P. falciparum*. The PfEMP1-mediated rosetting and endothelial cytoadhesion are two distinct biological phenomena, as demonstrated by previous studies (61, 162, 163). Nevertheless, dual cytoadhesion of rosetting IRBCs to endothelial cells have been demonstrated (164, 172), and distinct domain of PfEMP1 variant that possesses dual cytoadhesive (to endothelial cells and URBCs) properties has been described, albeit with very weak affinity to endothelia (the rosetting IRBCs were seen rolling instead of stably adhering to endothelial cells) under flow conditions mimicking microvasculature shear stress (164). Therefore, it remains to be investigated if such dual-binding phenomenon by IRBCs exists *in vivo*.

Importantly, all rosetting studies have been conducted under *in vitro* or *ex vivo* conditions using blood samples collected from peripheral circulation of patients, or clones of parasites derived from such sampling methods. The conundrum lies in the fact that the IRBCs that stably cytoadhere to microvasculature endothelium are responsible for parasite sequestration and may be the major contributor to the manifestation of severe malaria. However, the subpopulation of IRBCs collected from peripheral blood may be phenotypically different from those sequestering in the microvasculature when it comes to propensity of the IRBCs to cytoadhere. From another viewpoint, if the cytoadhesive phenotypes of IRBCs (usually the early stages) collected from the peripheral circulation are essentially similar to the sequestering late stage-IRBCs, the findings from *in vitro* rosetting studies (conducted on these parasites after *ex vivo* maturation) may not imply the actual situation *in vivo* since the recruited IRBCs are not given an equal exposure to URBCs and endothelial cells in rosetting assays, which raises doubts if rosetting ever happens *in vivo*. If this were the case, the rosetting phenomenon seen *in vitro* is merely an indication of “IRBC's stickiness,” where the IRBCs would probably adhere to the microvasculature wall *in vivo*. Such situation makes it difficult to extrapolate the importance of rosetting in contributing to pathogenesis of severe malaria.

Malaria pathogenesis develops with time and often takes days to occur. One of the important shortcomings of studies correlating severe malaria with cytoadhesive IRBCs is that these studies are essentially snapshots of a multi-step process, which may be difficult to capture the complete chronology of an infection's pathogenesis. For cases with low parasite density, IRBCs have plenty of endothelial cells to cytoadhere to, leaving the non-endothelial cytoadhering IRBCs available in peripheral circulation. To avoid splenic clearance, these IRBCs may default to form rosettes over IRBC-endothelial cytoadhesion. Hence, it would be difficult to draw any correlation between rosetting phenomenon by this IRBC subpopulation and the pathology development that is happening in the deep microvasculature. On the other hand, parasite density in certain patients from certain localities may become too high (depending on parasite's virulence, genetic background and immunity status of the host, or lack of accessibility to timely treatment) and over-saturated relative to the total surface area of deep vasculature endothelial cells available for IRBC cytoadhesion. Thus, the IRBCs that do not get to cytoadhere to endothelia will be available in peripheral circulation. Of note, the availability of late stage *P. falciparum*-IRBCs in peripheral blood of a patient suffering hyperparasitemia has been reported (173). When these IRBCs are collected for rosetting assay, they are provided with only URBCs. Without their preferred cytoadhesive target (endothelial cells), these IRBCs may form rosette with the URBCs. Such alternative binding may happen as host-derived receptors like heparan sulfate (HS) (26, 164, 174–176), and CD36 (28, 177) have been reported as the receptor for endothelial cytoadhesion and rosette formation by the IRBCs. Rosetting rates obtained from such samples may reflect the relative endothelial cytoadhesion propensity of the IRBCs, which is associated with the severe malaria development. This may explain the positive correlation between rosetting and parasitemia in African clinical isolates previously reported (168). This hypothesis may also partly explain the discrepancies in correlation studies of rosetting rates and malaria severity conducted in different parts of the world. Notably, earlier studies have shown that the parasite clones in peripheral circulation and those sequestering in deep vasculature are similar (178, 179). Nevertheless, these molecular findings were based only on MSP-1 and MSP-2 alleles, and the tissue tropisms of the parasite subpopulations in a patient may not be revealed without specifically analyzing genes related to cytoadherence, as highlighted by the study (179).

So, the question remains: does the rosetting phenomenon contribute to severe malaria apart from its role as an immune-evasion strategy? To date, there is still a lack of solid evidence demonstrating stable, direct binding of rosetting IRBCs to endothelial cells under flow conditions. Nevertheless, such event may still be possible if the site of occurrence (microvasculature) has its blood flow hampered significantly in advance by the IRBC-endothelial cytoadhesion. Alternately, the rosetting IRBCs may be adhered securely to the endothelial cells via platelet as elaborated earlier (147). Regardless of how the rosette-endothelial binding interactions are, the contribution by rosetting to vasculature occlusion may not even require direct cytoadherence of rosetting IRBC to the endothelial cells. As mentioned earlier, it was shown that rosettes are less deformable and takes longer time to flow through a capillary-mimicking micropipette (68). In addition, Kaul et al. (180) demonstrated in an *ex vivo* system using rat isolated mesocecum that rosetting IRBCs contributed to microvasculature occlusion under flow condition. In this system, rosette-forming *P. falciparum* IRBC formed aggregates at venule junction, which restricted the flow. These aggregates were eventually dissociated slowly by the induced upstream force mimicking blood flow, leaving some IRBCs still attached to the endothelial cells afterwards. Here, rosetting was seen as an event that “widens the zone of vasculature occlusion.” With merely IRBC-endothelial cytoadhesion, blockade may only happen at fine capillaries with lumen size (~5–10 μm) close to the size of a normal RBC. However, sites of IRBC sequestration encompass capillaries and venules (lumen size of ~ 7 μm to 1 mm) (73, 181). As pointed out by Nash et al. (68), even with a monolayer of IRBCs cytoadhering to its endothelial wall, venules should has lumen wide enough to allow circulation flow, albeit with higher resistance. Following this theory, rosetting may occlude microvasculature distal to the endothelial-cytoadhered IRBC-obstructed fine capillaries. Nevertheless, it is important to note that another species of human malaria parasite, *P. vivax*, also readily forms rosettes (182, 183). Besides, the rigidity of *P. vivax* rosettes also increases (94). However, *P. vivax*-related cerebral malaria cases are not as common, with majority of such cases being reported from India (184–190), suggesting involvement of the human host-derived factors in this relatively geography-restricted pathology. Importantly, the endothelial cytoadhesion phenomenon by *P. vivax* IRBCs has been demonstrated, which is of similar binding strength but ten times lower in frequency than that of *P. falciparum* IRBCs (191). Therefore, this suggests that the key player that drives vasculature occlusion is IRBC-endothelial cytoadhesion. In this context, rosette formation is likely to play a subsidiary role.

Genetic polymorphisms influencing rosetting receptor expression is another factor to consider when assessing the roles of rosetting in malaria pathogenesis. For example, low level expression of CR1 on the surface of URBC (receptor for both rosette formation and IRBC clearance by the host) was reported to be a risk factor for severe malaria in Thai population (192). Another polymorphism that increases RBC surface expression of CR1 was reported to confer protection against cerebral malaria development in Thai population (193). On the other hand, studies conducted in India yielded complex picture, where low CR1 expression was found to be correlated with severe malaria susceptibility in non-endemic regions whereas high CR1 levels were associated with disease development in the malaria-endemic areas (194). Another study conducted in eastern part of India reported that extremely high and extremely low expression level of CR1 can lead to the higher risk of cerebral malaria development (195). Likewise, studies from Africa and Papua New Guinea yielded conflicting outcomes (196–198). Recently, two distinct CR1 polymorphisms commonly seen in African populations were found to demonstrate opposing correlation with the development of cerebral malaria in Kenya (199). The *Sl2* allele was reported to confer protection against cerebral malaria, possibly due partly to its reduced rosetting phenomenon in addition to other factors, as suggested by the authors; whereas *Mc*C^*b*^ allele served as a risk factor to develop cerebral malaria, but arises from selection probably due to survival advantage against other infections (199). Based on the example above, it is not easy to draw clear conclusions based on the correlations between genetic polymorphisms in a population and the outcome of a *P. falciparum* infection. More downstream experiments with carefully controlled longitudinal studies are needed to validate the significance of these findings.

## Rosetting Against Endothelial Cytoadhesion?

Parasitism is a relationship between two organisms where one party (the parasite) causes harms to the other party (the host) while living in/on the host. Evolution, through selection process, tends to drive this relationship toward a relatively “peaceful” one, where the selected parasites cause as little harm as possible to the host while the host is evolved and adapted to accommodate the parasite, without eliciting much immune response against the parasites. Following this evolutionary point of view, it would make more sense that *P. falciparum* that do not kill its human host while trying to survive within its host would be selected over time. As elaborated earlier, the *P. falciparum* late stage-IRBCs require sequestration to escape host's immune system. However, the endothelial cytoadhesion-mediated sequestration causes potentially fatal outcomes to the host, which is disadvantageous to the parasite as well.

Importantly, in areas with seasonal malaria transmission, asymptomatic carriers of *P. falciparum* serve as the parasite reservoirs during dry seasons, when the *Anopheline* mosquito number is low (200–203). Parasites persist within the hosts for months without causing clinical symptoms. In addition, the severity of clinical presentations for falciparum malaria covers a broad spectrum. This suggests that sequestration of late stage-IRBCs away from peripheral circulation can still happen without inducing grave outcomes to the host. Is endothelial cytoadhesion the only way for the parasites to sequester and escape splenic clearance?

Interestingly, cytoadhesive events such as rosetting, autoagglutination, and endothelial cytoadhesion use PfEMP1 as their ligand. Is there any form of competition between these events *in vivo*? In fact, the whorl of URBCs around a rosetting IRBCs can serve as a mechanical barrier against IRBC-endothelial cytoadhesion (164, 204, 205) and autoagglutination (206). Does rosetting carry any merit in reducing or preventing the endothelial injuries? Such theory has been raised no long after the discovery of rosetting phenomenon, where the role of rosetting either as a friend or foe to human host relies on the location or timing of rosette formation (68). IRBC-endothelial cytoadhesion occurs at capillaries and venules. If rosettes are formed ahead of these sites, rosettes can prevent IRBC-endothelial cytoadhesion. If rosettes can only be formed at similar vasculature sites as the IRBC-endothelial cytoadhesion, rosettes formed by the already endothelial-cytoadhered IRBCs can worsen the vasculature occlusion.

The manifestation of rosetting relies on the stability of rosetting complex under flow conditions. Rosettes are stable under sheared conditions, from very low shear forces to shear stress of about 1.5 Pa (68, 207), which is applicable to shear stress generated by blood flowing through arteries (208). This suggests that rosettes are available throughout the systemic circulation and that the *in vivo* rosettes may prevent IRBC-endothelial cytoadhesion. One concern was raised by an earlier study based on observation from its micropipette assay (68), where a rosetting IRBC that is forced into a capillary by blood flow will eventually have direct contact with the capillary wall (endothelial cells), hence IRBC-endothelial cytoadhesion may still happen even with rosetting. It is important to note that the force applied by that study to maneuver the rosetting IRBC into the micropipette was much higher (30 Pa) than the *in vivo* arterial shear force. Assuming that rosettes cannot move into capillaries *in vivo*, they may block the flow of blood into the capillary bed. If this were the case, the brain tissues covered by the affected capillary bed would suffer hypoxia and irreversible damages. However, cerebral malaria cases with irreversible hypoxia-induced brain tissue damages (as in stroke patients) following microvasculature occlusion by the IRBCs are rarely seen (209). Interestingly, via microvasculature-mimicking microfluidics channels, it was observed that the more rigid *P. vivax* rosettes that blocked the channel openings did not occlude the flow of normal URBCs through the channels (94). Although the experiment was conducted with *P. vivax*, we believe that it is applicable to *P. falciparum* as well, since both species preferably rosette with normocytes (matured RBCs) with similar binding strength (94, 183), and the rosettes formed by both species show enhanced rigidity (68, 94).

Another interesting evidence that suggests rosetting as “counter-endothelial cytoadhesion” stems from studies that investigated effects of sulfated glycoconjugates on rosetting and IRBC-endothelial adhesion. A number of sulfated glycoconjugates such as fucoidan, dextran sulfate, and heparin can disrupt rosettes (174, 210, 211). However, these molecules were found to enhance cytoadherence of IRBCs to CD36-bearing endothelial cells (177). An earlier study also reported the need of rosette disruption to allow IRBC adherence to CD36 (205). These findings suggest the need of cautious approach in considering heparin-derived molecules as malaria adjunctive treatment on the ground that they can disrupt rosette formation, as such adjunctive therapy may worsen the clinical situation by promoting IRBC-endothelial cytoadhesion (177). Is rosetting by the IRBCs purely a risk factor to human host, or an attempt by the parasites to minimize damages to the host without compromising its own survival? Various host- and parasite-derived confounding factors complicate the role characterization of rosetting.

## Involvement of Human-Derived Factors in Shaping the Direction of IRBC-Cytoadherence?

To date, most of the studies on human host-malaria parasite interactions in the context of IRBC cytoadherence focus on injuries sustained by the host from the parasites. Whether there is any “damage control” approach by either party in this parasitism relationship remains unknown. This is rather bizarre for a parasitism relationship with such a long evolutionary history. Importantly, as mentioned earlier, the parasites can persist in some human hosts for a very long time without causing signs and symptoms. This suggests that the survival-essential phenomena of the parasites, such as deep vasculature sequestration to avoid splenic clearance, can be tolerated by the host, and these phenomena may be the result of host-parasite interactions. Interestingly, the host-derived complement factor D, albumin, and anti-band 3 IgG have been reported as the rosette-promoting factors for *P. falciparum* (212). On top of that, a recent study reported “something” other than IgG from pooled human sera inhibited cytoadhesion of PfEMP1 to EPCR (213). Such serum-mediated IRBC-cytoadherence inhibition suggests intervention attempts by the host to control damages. According to this study, the inhibitors are available in circulation even under non-malaria infected conditions (usage of pooled donor sera) (213). Nevertheless, it is not known if there is any underlying medical condition among the donors. This is important since the serum component profiles of individual with cardiovascular problems, diabetes or chronic subclinical inflammation may be different from those of optimal health condition (214, 215). In addition, the components of serum from peripheral blood maybe different from that of the microenvironment within deep vasculature suffering endothelial injuries following IRBC-endothelial cytoadhesion. Nevertheless, this study sheds lights on potential host-parasite interactions in malaria pathogenesis.

As stated earlier, the adherence of IRBCs to endothelial cells trigger endothelial activation and inflammation. Subsequently, the level of various cytokines at the inflamed site is increased. Weibel-Palade body is one of the components being released by endothelial cells upon the onset of endothelial activation. As elaborated earlier, of the various components found within Weibel-Palade bodies, some have been associated with severe malaria pathogenesis and some have important role in regulating homeostasis in vasculature. It would be interesting to examine the effects of all key components in Weibel-Palade bodies on the dynamics of IRBC cytoadherence, as well as other interplays between the host and the parasite.

**Figure 2:**
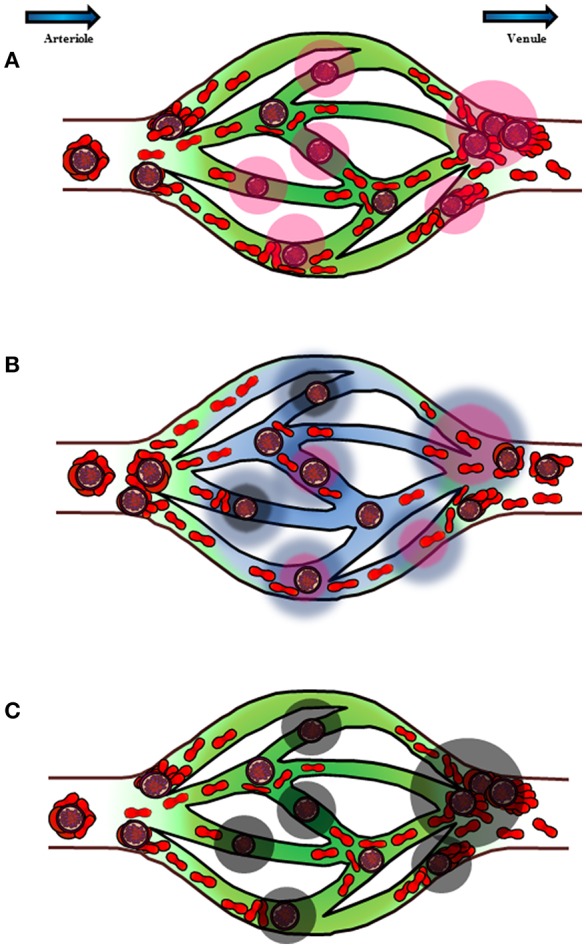
Schematic diagram to illustrate the postulated chronology and mechanism of *P. falciparum* sequestration and pathogenesis in deep vasculature. The blue arrows on top of the diagram represent the direction of blood flow from arteriole to venule. **(A)** Cytoadhesion of IRBCs on endothelial cells causes endothelial inflammation. In addition, rosette formation at the capillary junctions opening into venules also contributes to the hampering of blood flow within the vasculature. The endothelial inflammation by direct IRBC-cytoadherence, coupled with hampering of blood flood stimulate the affected endothelial cells (pink halo) to release various substances in response to the changes in its environment. **(B)** Some of the components released by the endothelial cells (blue halo) may reverse and prevent IRBC-endothelial cytoadhesion, at the same time stimulate rosette formation. Rosetting mechanically prevents IRBCs from binding to endothelial cells while enabling the IRBCs to sequester in larger microvasculature. This will enable the parasite to escape splenic clearance. This switch of cytoadhesive characteristics also prevents complete occlusion of blood flow, thus minimizing, if not preventing irreversible tissue damages from tissue hypoxia. **(C)** However, for hosts with endothelial cells that are not as well-responsive to IRBC-endothelial cytoadhesion and slowing down of blood flow, the components that can reverse and prevent IRBC-endothelial cytoadhesion may be inadequate to exert such effect. As a result, vasculature occlusion ensures. At the same time, endothelial injury and vasculature leakage worsen (black halo), which may lead to fatal outcome.

Based on the currently available literature, it is likely that the malaria-related cytoadherence phenomena may give rise to complex host-parasite immunopathological interactions, starting from IRBC-endothelial cytoadhesion at the microvasculature (Figure 2). This results in endothelial activation and vasculature inflammation. Blood flow slows down due to the IRBC sequestration, which enables some immediately reformed rosettes with dual cytoadhesive capability to adhere at the venular junction. In fact, vasculature areas subjected to complex shear stresses such as the vascular branching junctions have abundant VWF-containing Weibel-Palade bodies (144), which may facilitate IRBC-endothelial binding. This further aggravates vasculature occlusion. On the other hand, stable rosettes that are formed before entering capillary bed will stay at the arteriole due to the higher rigidity of the whole rosetting structure. This form of rosettes prevents the rosetting IRBCs from having direct contact with the capillary endothelial, hence preventing endothelial cytoadhesion. Meanwhile, the reduction in shear stress of the microvasculature (capillaries and venules), coupled with endothelial inflammation trigger the affected endothelial cells to alter their expression, releasing unknown factors that may reverse IRBC-endothelial cytoadhesion. The endothelial cells may also secrete some other components to shield the endothelia from cytoadherence by incoming IRBCs, or the aforementioned unknown factors may be capable of reversing and preventing IRBC-endothelial cytoadhesion. The IRBCs detached from endothelial cells will flow out of the capillary bed into the venule, and form rosettes. The rigid structure of rosettes enables the IRBCs to escape splenic clearance by mechanically sequester in larger-size microvasculature. This may avoid further damaging of host's vasculature, minimize complete occlusion of blood flow hence hypoxia and tissue necrosis. Finally, the ability of the host to respond to IRBC-induced endothelial activation by secreting and releasing these anti-endothelial cytoadhesion mediators will determine his survival in battling malaria.

## Concluding Remarks

Undeniably, IRBC cytoadhesion is an important aspect in the pathogenesis of malaria, and a key interplay between the malaria parasite and its host. However, there are many “unresolved issues,” such as the role of rosetting and feedback responses by the host following malaria-induced vascular injury, which deserve further research attention. A better understanding on these issues will enable us to understand malaria pathogenesis better, and design a reliable and safe clinical intervention strategies to improve the clinical management of malaria patients.

## Author Contributions

All authors listed have made a substantial, direct and intellectual contribution to the work, and approved it for publication.

### Conflict of Interest Statement

The authors declare that the research was conducted in the absence of any commercial or financial relationships that could be construed as a potential conflict of interest.
